# Time Course of Recovery for Performance Attributes and Circulating Markers of Muscle Damage Following a Rugby Union Match in Amateur Athletes

**DOI:** 10.3390/sports8050064

**Published:** 2020-05-18

**Authors:** Bruno Victor Corrêa da Silva, Mário Antônio de Moura Simim, Rodrigo Barboza da Silva, Edmar Lacerda Mendes, Bernardo Neme Ide, Moacir Marocolo, Jeffrey S. Martin, Gustavo R. Mota

**Affiliations:** 1 Exercise Science, Health and Human Performance Research Group, Department of Sport Sciences, Institute of Health Sciences, Federal University of Triangulo Mineiro, Uberaba 38025-350, Brazil; brunobjjbh@gmail.com (B.V.C.d.S.); rodrigobarboza85@gmail.com (R.B.d.S.); edmar.mendes@uftm.edu.br (E.L.M.); grmotta@gmail.com (G.R.M.); 2 Department of Environmental, Biological and Health Sciences, University Center of Belo Horizonte (Uni-BH), Belo Horizonte 30575-180, Brazil; 3 Research Group in Biodynamic Human Movement, Institute of Physical Education and Sports, Federal University of Ceará, Fortaleza 60020-181, Brazil; 4 Department of Biochemistry and Tissue Biology, Institute of Biology, State University of Campinas, Campinas 13083-970, Brazil; bernardo_311@hotmail.com; 5 Physiology and Human Performance Research Group, Department of Physiology, Federal University of Juiz de Fora, Juiz de Fora 360360-900, Brazil; isamjf@gmail.com; 6 Department of Basic Medical Sciences, DeBusk College of Osteopathic Medicine at Lincoln Memorial University—Knoxville, Knoxville, TN 37932, USA

**Keywords:** rugby, muscle damage, change of direction, acceleration, speed, recovery, team sports, performance, time course, testing

## Abstract

Background: We sought to determine the time course of changes in neuromuscular performance and muscle damage following a single rugby union match. Methods: Fourteen male amateur rugby players (28.9 ± 3.5 yrs; 1.7 ± 5.1 m; 86.1 ± 11.1 kg) participated. Plasma activity of creatine kinase ([CK]) and lactate dehydrogenase (LDH), L-run test (change of direction) and 30-m sprint (T30; speed) with 10-m lap time (T10; acceleration) were assessed on six occasions: one week before the match (PRE) and immediately, 24, 48, 72, and 96 h post-match. Results: Relative to PRE, LDH was elevated immediately post-match (+33.6% ± 13.6%; *p* < 0.001) and [CK] was elevated immediately (+64.1% ± 38.8%, *p* = 0.001) and 24 h post-match (+352% ± 317%; *p* = 0.024). L-run test time increased 16.0 ± 8.7% relative to PRE at 24 h post (*p* < 0.001) and remained elevated through 96 h post-match (*p* < 0.05). T10 and T30 times increased relative to PRE immediately post-match (+12.0% ± 10.4%, *p* = 0.008; and +6.1% ± 4.9%; *p* = 0.006, respectively), though T30 times were similar to baseline by 48 h post-match whereas T10 times remained elevated through 72 h post-match. Conclusions: A single, competitive rugby union match induces significant muscle damage and performance decrements with distinct time courses of recovery in amateur athletes. Notably, change of direction attributes (i.e., L-run) appear to have the longest time course to full recovery.

## 1. Introduction

Rugby is a body contact sport played across different competitive levels (e.g., amateur to professional). A typical match consists of two 40 min halves separated by 10–15 min, with frequent intense bouts of high-intensity activities such as running, kicking, passing, and tackling interspersed with low-intensity exercise [1]. Furthermore, during a match rugby players perform a high number of accelerations and decelerations with change of direction involving eccentric muscle actions [2]. Because of these demands, significant skeletal muscle fatigue and muscular damage are reported after a rugby match [3].

Muscle damage after competitive rugby matches is well reported in the literature, mostly described by changes in creatine kinase ([CK]), myoglobin, and lactate dehydrogenase (LDH) [1]. These indirect markers of skeletal-muscle damage have demonstrated different responses after a rugby match. Myoglobin and LDH concentrations have been shown to peak 10–45 min after a match, [4,5], whereas [CK] has been shown to peak at ~24 h post-match [4] while remaining elevated for up to 120 h [6]. Notably, the degree of rugby match-induced muscle damage is related to the number of physical contacts and by the total number of high-intensity accelerations and decelerations [7].

While elevations of the aforementioned blood markers of muscle damage are indicative of myofibrillar disruption, it does not indicate the magnitude of impaired muscle function [8]. With this in mind, combining [CK] measures with other indirect markers increases the reliability and interpretations of the muscle damage and fatigue markers associated with a rugby match. Indeed, post-match neuromuscular function reductions can persist despite normalized blood markers of muscle damage [9].

With regards to neuromuscular function, a rugby match induces impairment in countermovement jump performance [3], interruption of peripheral contractile function, and voluntary torque suppression for up to 4 days post-match [3,6]. Although, the vertical jump reflects stretch-shortening capability of the lower-limb and isokinetic and isoinertial dynamometry are able to provide data on isolated muscle groups, the ecological validity of these tests may be poor because they do not totally replicate the main specific rugby movements [10]. Thus, the use of tests that replicate the movement pattern or physiological demands of the team sport to detect neuromuscular recovery has been suggested [3,11,12]. To the best of our knowledge, only one study specific to rugby has employed this strategy. The results of that investigation related a decrease in acceleration into the contact zone after a repeated-effort protocol designed to reflect the extreme demands of competition. Furthermore, a relationship between change of direction speed and 10-m sprints with decrement in tackling technique under the fatigued condition was found [2].

Although the impact from training and competition on fatigue in rugby has been reported extensively in the scientific literature [3], there is no information regarding a comprehensive time course of recovery for change of direction attributes or sprint performance after a single amateur rugby match. Given that muscle damage and fatigue post-match may differ between professional and amateur athletes due the difference in fitness [13], a longer time course evaluation (e.g., ≥96-h) for recovery is important for practical applications. Additionally, insufficient recovery, with ensuing fatigue could potentially lead to poor or inconsistent performances and injury, particularly in amateur players [3]. Thus, this study aimed to evaluate the time course of the neuromuscular responses using more sport-specific tests (speed and change of direction runs) and indirect muscle damage markers (plasma [CK] and LDH) for 96 h after a single amateur rugby union match.

## 2. Materials and Methods

**Figure 1:**
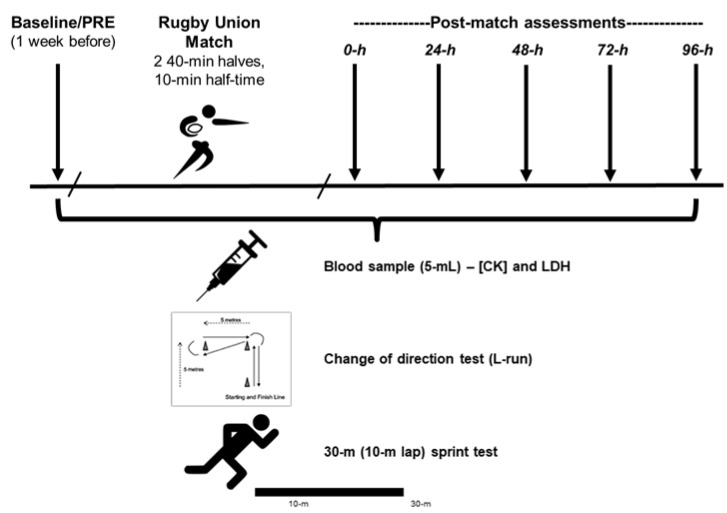
Experimental design. [CK], creatine kinase; LDH, lactate dehydrogenase.

Neuromuscular performance and muscle damage markers were assessed before and for 96 h following a single, competitive rugby union match. Specifically, neuromuscular performance (speed/change of direction tests) and blood samples were assessed 1 week before (PRE) and at five times following the match; immediately post (Post/0 h) and 24, 48, 72, and 96 h post-match (Figure 1). In the week before and after PRE measurements, the participants were instructed to participate in only light training sessions/practice on 2–3 occasions. We assessed the PRE measurements 1 week prior to the match because of team logistics and schedules, and because one week was allowed for a significant recovery period to minimize interference with muscle damage markers and neuromuscular fatigue from the physical tests themselves [3,6]. The PRE measurements were done at ~17:50 with subsequent measurements scheduled at approximately the same time each day (match finished at ~17:30) in an effort to avoid differences due to diurnal variation. The order for all participants’ data collection (i.e., blood samples and physical tests) were the same across all time points and players were given no information about their data (e.g., time/performance on the tests) during the study to prevent bias [14]. During the days after the match, participants were instructed not to perform any physical exercise or engage in any formal recovery processes (e.g., stretching and massage).

Performance was assessed using the L-run test (change of direction attributes) and the 30-m running sprint test (T30) with a lap time at 10 m (T10; speed and acceleration). We used these tests given their similarity with several actions of the rugby game as well as their utility in measuring acceleration, maximum speed, and change of direction attributes of the players [15]. Given that these tests were performed on a regular basis throughout the season, all players were familiar with them prior to study enrollment. Muscle damage was assessed by plasma [CK] and LDH activities. 

### 2.1. Participants

Fifteen male, amateur rugby union players participated in this study, but one was excluded because of the absence of his time-course measurements. Thus, 14 players (N = 14) completed the entire study (six backs and eight forwards). On average, players were aged 28.9 ± 3.5 yrs, had a height of 1.7 ± 0.5 m, and had a body mass of 86.1 ± 11.1 kg. All participants were considered healthy and were engaged in training and competition in the state league schedule for at least 6 months. The Federal University of Triangulo Mineiro ethics committee for human research approved this study before any data collection (approval number 279.2116). The study was conducted in accordance with the Declaration of Helsinki. Participants were informed about the benefits and risks of the investigation, and they voluntarily signed an informed consent document prior to their participation.

### 2.2. Procedures

#### 2.2.1. Plasma Activity of Muscle Enzymes

Before the physical tests, blood samples were collected from the antecubital vein in a 5 mL EDTA tube (BD Vacutainer^®^, Franklin Lakes, NJ, USA) for subsequent analysis of plasma markers. On each day of collection, blood samples were centrifuged at 1008 g to separate plasma from other blood constituents. Plasma samples were then transferred into Eppendorf tubes and stored for future analysis of [CK] and LDH by kinetic UV method, using two hematology analyzers: Cobas Mira Plus (Roche Diagnostic Systems, Welwyn, UK) for [CK], and Cobas Integra 400 Plus (Roche Diagnostics, Rotkreuz, Switzerland) for LDH.

#### 2.2.2. L Run Test

With the exception of the one measurement occurring immediately following the rugby match, prior to measuring change of direction run and sprint performance, all players performed a 12 min standardized warm-up. The warm-up consisted of 7 min running (~60% heart rate max) and 5 min of intermittent straight runs of ~10-m, including maximum accelerations, interspersed with ~100-m of low intensity running. 3 min after warm-up, three cones were placed in an “L” shape, 5-m apart. Positioned at the same line of the first cone, after the sound signal (horn), the player ran forward, turned to the left at the second cone, and went toward the third and last cone, returning by the same route to the starting line. Players were instructed to complete the route as rapidly as possible. Running time was recorded using a photocell system (Speed Test 6.0 Tel CEFISE^®^, São Paulo, Brazil) located at the start and finish line. Time for the faster of two attempts with 2 min of passive rest in-between was recorded.

#### 2.2.3. 30-m Sprint Test (T10 and T30)

Three min after the L-run test, each player performed a maximal 30-m sprint. After an audible signal (whistle), they ran 30-m straight as fast as possible. Time for T10 and T30 was recorded electronically using the same photocell gates system, with one photocell located 10-m from the starting line and another located 30-m from the starting line. Three attempts were made with 3 min rest between successive attempts and the fastest times (T10 and T30) were recorded [16]. T10 was measured to estimate the acceleration time. T30 was measured to estimate speed and selected based on previous investigations [17,18] in which it was found that the average duration of rugby union match sprints varied between 2.01 and 3.84 s.

#### 2.2.4. Rugby Union Match and Rate of Perceived Exertion

The match was performed at 16:00 on a regular rugby union field (size, 100-m × 70-m; temperature, ~23 °C and humidity, ~40%) following the official union rules (15 players each team; two halves of 40 min each with a 10 min half-time). The match used for this study was a semi-final of an amateur state championship. The participants in the current study belonged to one of the competing teams. After the match, each player indicated a score for their rating of perceived exertion (RPE) via the CR-10 Borg scale (ranges from 0 to 10, where 0 is “nothing at all” and 10 is “very, very hard; maximal”) to determine the subjective, personally perceived intensity of the match [19]. The players reported their respective score individually to prevent potential influence on/from another player [20,21].

### 2.3. Statistical Analyses

The Shapiro Wilk test was employed to verify the normality of data. The normally distributed data were analyzed by repeated measures analysis of variance (RM ANOVA—time factor) with Bonferroni correction for multiple comparisons. Significance was set at an alpha value of 0.05 and *p*-values reported herein are the Bonferroni corrected values. Statistical analyses were performed using IBM SPSS Statistics 26 for Windows (Chicago, IL USA). Data in figures are presented as mean ± standard deviation and data in the results text are presented as mean effect size with 95% confidence interval [lower bound, upper bound]. 

## 3. Results

### 3.1. Rugby Union Match And Rating of Perceived Exertion

All participants performed the whole match (no substitutions). After the match, the mean rating of perceived exertion for all players was 9.9 ± 0.4 (10-pt scale).

### 3.2. Plasma Activity of Muscle Enzymes—Creatine Kinase ([CK]) and Lactate Dehydrogenase (LDH)

**Figure 2:**
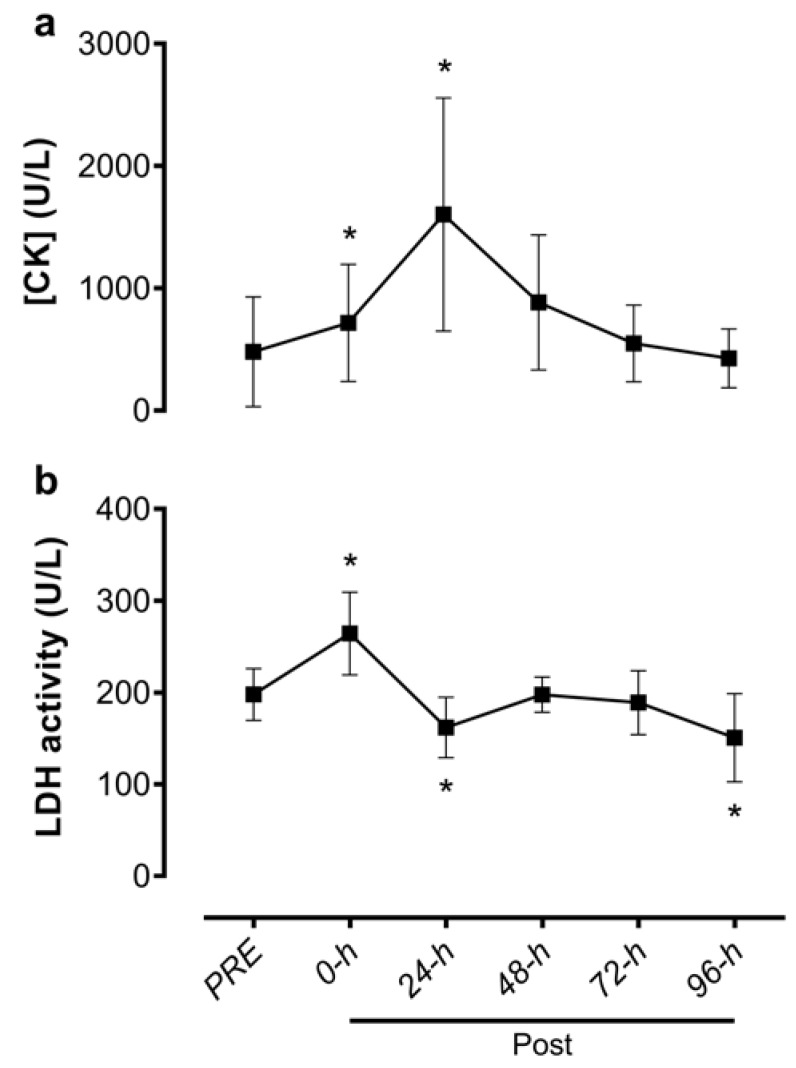
Plasma activities of (**a**) creatine kinase ([CK]) and (**b**) lactate dehydrogenase (LDH) 1 week prior to the rugby match (PRE), immediately post-match (Post) and 24, 48, 72, and 96 h post-match. Data are presented as mean ± standard deviation. *, significantly different from PRE values (*p* < 0.05).

There was a significant effect of time for plasma activity of both [CK] (F_5, 65_ = 13.5; *p* < 0.001) and LDH (F_5, 65_ = 26.8; *p* < 0.001). Plasma [CK] activity was significantly elevated relative to PRE at the immediate post (+64.1% [+25.3%, +103%], *p* = 0.001) and 24 h post-match (+352% [+35.1%, +669%], *p* = 0.024) time points, but not different thereafter (Figure 2a). Plasma LDH activity was significantly higher than PRE values at the immediate post-match time point (+33.6% [+19.9%, +47.4%], *p* < 0.001) and significantly lower at the 24 (−17.9% [−31.8%, −3.9%], *p* = 0.008) and 96 h post-match (−24.3% [−42.8%, −5.8%], *p* = 0.006) time points (Figure 2b).

### 3.3. L Run Test

**Figure 3:**
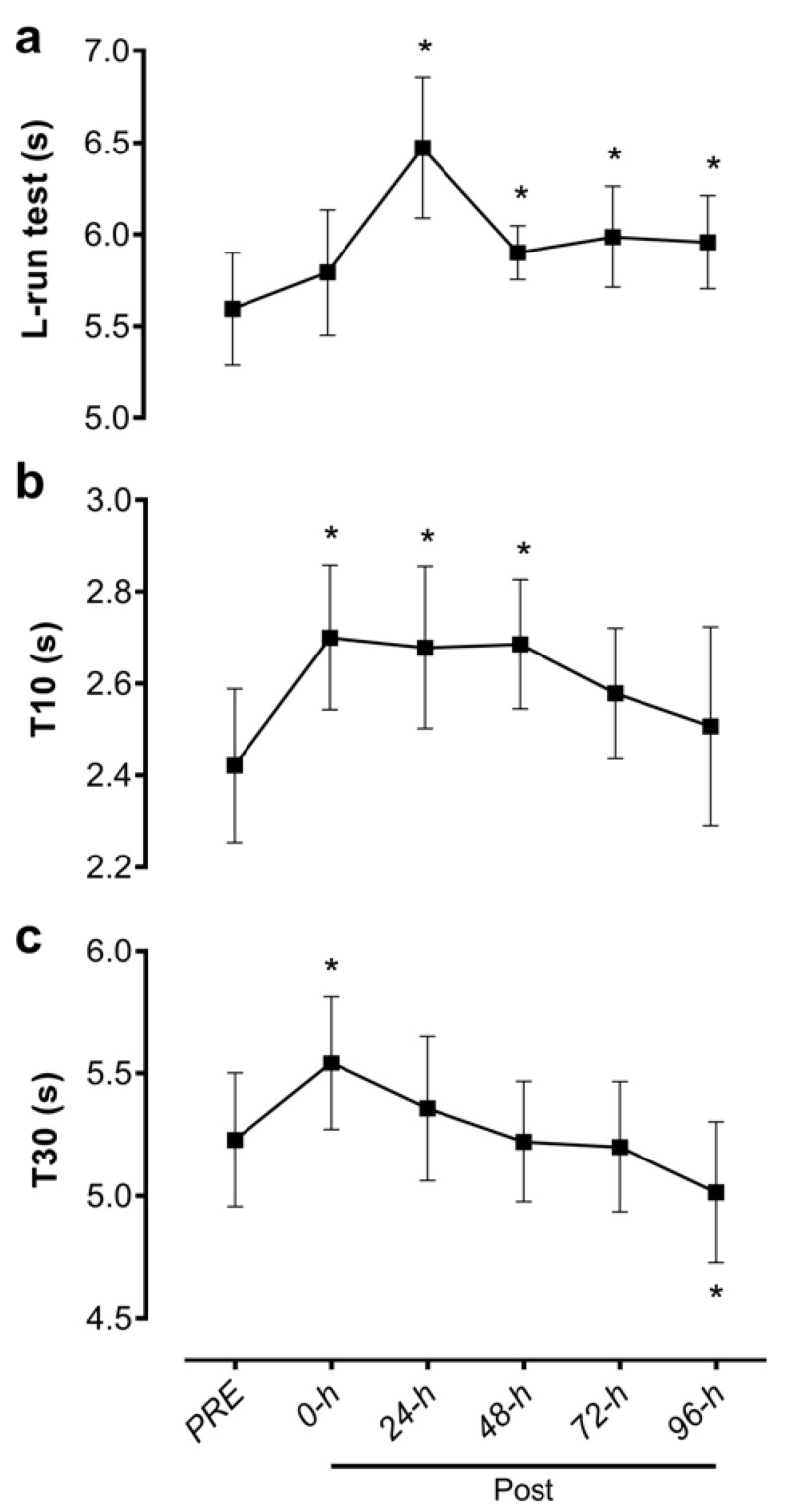
Result times for (**a**) L run test, (**b**) 10 m sprint (T10) and (**c**) 30 m sprint (T30) 1 week prior to the rugby match (PRE), immediately post-match (Post) and 24, 48, 72, and 96 h post-match. Data are presented as mean ± standard deviation. *, significantly different from PRE values (*p* < 0.05).

There was a significant effect of time for the L run test time (F_5, 65_ = 23.5; *p* < 0.001). L run tests times were significantly longer compared to PRE at the 24 (+0.88 s [+0.46 s, +1.30 s], *p* < 0.001), 48 (+0.31 s [+0.01 s, +0.61 s], *p* = 0.049), 72 (+0.39 s [+0.07 s, +0.72 s], *p* = 0.011), and 96 h (+0.36 s [+0.06 s, +0.67 s], *p* = 0.012) post-match time points, but were not different from PRE at the immediate post-match time point (+0.20 s [−0.01 s, +0.41 s], *p* = 0.061; Figure 3a).

### 3.4. Sprint Tests (T10 and T30)

There was a significant effect of time for both the T10 (F_5, 65_ = 10.5; *p* < 0.001) and T30 (F_5, 65_ = 13.4; *p* < 0.001) sprint tests. Relative to PRE, T10 time was significantly slower immediately post-match (+0.28 s, [+0.06 s, +0.50 s]; *p* = 0.008) and remained slower at 24 (+0.26 s, [+0.33 s, +0.48 s]; *p* = 0.019) and 48 h (+0.26 s, [+0.09 s, +0.44 s]; *p* = 0.001) post-match (Figure 3b). T30 was also significantly slower relative to PRE immediately post-match (+0.31 s, [+0.08 s, +0.55 s]; *p* = 0.006), but returned to PRE levels by 24 h and were significantly faster at 96 h post-match (−0.21 s, [+0.33 s, +0.10 s]; *p* = 0.006; Figure 3c).

## 4. Discussion

This study aimed to evaluate the time course of neuromuscular performance and muscle damage after a competitive rugby union match. Our main findings were that a single, competitive rugby union match in amateur athletes induces muscle damage as indicated by plasma LDH and [CK] activities, and decrements in physical performance, especially in short distance sprint (i.e., 10-m) and change of direction activities (both persisting for a greater period of time than 30-m performance decrements). To the best of our knowledge, this is the first study to explore neuromuscular response with a comprehensive time course of recovery (i.e., up to 96 h) using sprint and change of direction tests, after a competitive rugby match, providing valuable practical information to coaches and sports scientists.

The current data were collected after an official semifinal amateur competitive match (state level), which is more stressful than simulated rugby matches [22,23]. Because of this competitive scenario, we expected a very high intensity and the 9.9/10 RPE we observed confirmed this expectation [24]. A particular strength of our data is that they were collected in the context of a real match, thus ensuring specificity to the event under investigation. Such validity is vital in sports science investigations [23,25].

Performance in the T10 and T30 sprint decreased post-match. Specifically, the 10-m sprint performance remained depressed longer, until 48 h post-match (Figure 3b), while the 30-m sprint showed decrement only immediately following the match (Figure 3c). Interestingly, the depression in change of direction run performance (i.e., increased L-run time) was even more pronounced than sprint performance, and it remained depressed for at least 96 h post-match observed in this study. This implies that rugby-induced muscle damage and fatigue [3,5] probably affect the neuromuscular ability to accelerate, decelerate, and change directions quickly to a greater extent than straight-line running. This information can help coaches to better organize their training program after matches, especially in the few days following a game. In fact, these findings corroborate reports of lower indicators of muscle function (e.g., peak force, power output, vertical jump height) post-match in rugby players [13]. Also, a decrement in the change of direction tests has been found after basketball games [26], soccer small-sided games [27], and soccer matches [28,29].

The [CK] and LDH responses found post-match are similar to previous findings of rugby [6,7,22,24] and other team sports [30]. It has been demonstrated that [CK] presents a slow increase and reaches peak values at ~24 h post-match and continues to be elevated until 48–96 h post-match [6,22]. On the other hand, LDH peak values occur immediately post-match with a return to baseline at ~48 h post-match [4]. Herein we did observe peaks in [CK] and LDH consistent with those previously reported (LDH immediately post-match, [CK] at 24 h post-match). However, return to PRE values was faster for LDH (≤24 h post-match) than has been previously reported. Nevertheless, the differences in the time course for peak and recovered [CK] and LDH plasma activities could be due to the differences in protein efflux and/or clearance rate between [CK] and LDH. Indeed, efflux may be different due to the location and/or binding characteristics at which these enzymes are sequestered within the muscle sarcomere and dependent upon the site of primary mechanical muscle damage [31]. The efflux of [CK] and LDH proteins from muscle has been attributed to increased permeability of the plasma membrane and/or intramuscular vasculature. This increase in LDH and [CK] in blood after rugby match is, in part, a consequence of the number of physical contacts performed during the game and the high volume of intermittent running [3,7]. Indeed, the direct impact of tackles on the body is considered a primary cause of muscle damage observed after a rugby match [7]. Moreover, repeated eccentric muscle actions involved in intermittent sprint running during the landing phase, and to rapidly slowing the body (deceleration) contribute to higher magnitudes of muscle damage [13].

Besides the temporal difference, different quantitative responses have also been observed between [CK] and LDH. In the present study, the peak values of [CK] exceed 1000 U/L, which are similar to or greater than values reported by other studies following rugby efforts [5,6]. However, the peak LDH values (264.2 ± 45.1 U/L) we observed immediately following the rugby match were below those previously reported for Japanese rugby players (519 ± 79.2 U/L) at the same time point [4] and may explain the more rapid return to baseline we observed. This discrepancy may be due to differences in the experimental design of the studies. For example, the fluid for measurement (blood sample vs. interstitial fluid), fitness level, position of players and the unique characteristics and intensity of each game [13] may have influenced the results.

The absence of match measurements (i.e., time-motion analysis) is a limitation of the current study, which could explore other factors such as total distance covered, distances covered at high velocity, and the number of sprints. Moreover, responses could vary by position (e.g., forward vs. back) though the sample size of the current investigation limits the power for such analyses. Finally, in practice it is likely that athletes will perform some additional activities (e.g., travel, light exercise, myofascial massage) which may have an impact on the time course of recovery during the 96 h post-match. Herein, our controlled recovery environment speaks to recovery times devoid of therapeutic intervention.

## 5. Conclusions

We conclude that a single rugby union match induces significant reductions in neuromuscular performance with distinct time courses of recovery and muscle damage over time, suggesting an accumulated fatigue post-match. Performance was especially affected for acceleration (short distance sprint time [10-m run]) and change of direction attributes (L-run test), the latter of which was affected for a longer duration. Thus, full performance recovery (including acceleration and change of direction attributes) may require an extended recovery period (i.e., at least 96 h) that is beyond recovery of plasma markers of muscle damage and longer distance straight-line sprint performance.
